# Advancing Human–Animal Interaction to Counter Social Isolation and Loneliness in the Time of COVID-19: A Model for an Interdisciplinary Public Health Consortium

**DOI:** 10.3390/ani11082325

**Published:** 2021-08-06

**Authors:** Angela M. Hughes, Lindsey Braun, Alison Putnam, Diana Martinez, Aubrey Fine

**Affiliations:** 1 Mars Petcare, 18101 SE 6 Way, Vancouver, WA 98683, USA; 2 Human Animal Bond Research Institute (HABRI), 1310 L St, NW, Suite 860, Washington, DC 20005, USA; lbraun@habri.org; 3 Humane Rescue Alliance, 71 Oglethorpe St NW, Washington, DC 20011, USA; aputnam@humanerescuealliance.org; 4 Department of Education, College of Education and Integrative Studies, California Polytechnic State University Pomona, 3801 W. Temple Ave, Pomona, CA 91768, USA; dmartinez1@cpp.edu (D.M.); Ahfine@cpp.edu (A.F.)

**Keywords:** social isolation, loneliness, COVID-19, human–animal interaction, human–animal bond, leadership consortium

## Abstract

**Simple Summary:**

The value of the human–animal bond to human health and well-being, especially in a post-pandemic world, merits further study and advocacy. Social isolation and loneliness are widespread and exact a heavy toll on human physical and mental health, a burden worsened by the social distancing and quarantine measures of COVID-19. Interaction between humans and animals, whether experienced in the home or through animal-assisted therapy, has been shown to ease loneliness and lessen social isolation. This article describes how an innovative multidisciplinary partnership of leaders in the area of human–animal interaction—from veterinary science to nonprofit organizations and the pet care community—came together to advance research and practice, as well as tackle barriers in this promising area. It describes the concrete results already achieved by the initiative and offers a roadmap for others seeking to bring together diverse stakeholders to address issues and unmet needs through a similar collaborative model (herein called a consortium).

**Abstract:**

The mental and physical human costs of social isolation and loneliness—and their possible amelioration through human–animal interaction (HAI)—have both received intense attention since the onset of the COVID-19 pandemic, and its lockdowns, quarantines, and related mitigation measures. Concern about society’s “loneliness epidemic”, however, predates the pandemic, as does serious inquiry into HAI as a positive intervention. Recognizing the potential of companion animals to make a difference on an important public health issue, the Consortium on Social Isolation and Companion Animals—a novel partnership of the Human Animal Bond Research Institute (HABRI) and Mars Petcare—launched a joint initiative in 2019 to advance HAI research, address barriers to HAI, and support best practices in bringing together animals and people to ease loneliness. Beginning with a first-ever summit of multidisciplinary thought leaders, this collaboration has already yielded actionable insights and research projects. As a novel partnership initiative in the HAI field, it offers a promising model for future cross-disciplinary forward thinking to elevate HAI for the mutual benefit of companion animals and their welfare, as well as vulnerable human populations.

## 1. Introduction

Isolation and loneliness constitute an epidemic of our times, one brought to renewed attention by a global pandemic. Loneliness has been described as a debilitating psychological condition, one marked by a sense of emptiness, lack of control, low self-worth, and personal threat [1]. Social isolation, a contributor to loneliness, but not its sole determinant, refers to the absence of social interactions, contacts, and relationships with family and friends, neighbors, and society at large.

Whereas social isolation denotes a scarcity of connections or interactions, loneliness is the subjective perception of isolation—the discrepancy between an individual’s desired and actual level of social connection [2]. Indicators of social isolation include living alone, having a small social network, participating infrequently in social activities, and loneliness itself [3]. Social support, a related concept, includes the care and resources (emotional, physical, and financial) provided by others via social connection [2].

The prevalence of loneliness is high, varying among populations depending on factors including country of origin and measurement used [4]. Old age is commonly perceived to be associated with loneliness, but self-assessed loneliness actually decreases with age in some reports. Social support, meaningful daily interactions, and low social anxiety have been more strongly associated with decreased loneliness than structural or demographic factors such as age [5].

Nonetheless, social isolation and loneliness afflict millions of older adults who weather stressful life transitions and health issues, and for whom the physical health risks of loneliness may be especially severe [3]. Large surveys conducted by AARP, a US-based interest group focused on issues affecting those aged >50 years, indicate that about one-third of US adults aged ≥45 report feeling lonely, their numbers growing as the population of older adults increases [6]. Other populations vulnerable to the adverse impact of loneliness include adolescents [7], and individuals living with chronic physical or mental health challenges [4].

The present article reviews the evidence for human–animal interaction (HAI) as a resource to ameliorate social isolation and loneliness, describes how a multidisciplinary leadership consortium tackled this public-health challenge by creating a summit event and dedicated working groups to advance HAI, and presents a road map for others seeking platforms for fostering forward thinking, activism, and positive change.

## 2. Loneliness as a Public Health Crisis

Decades of research confirm loneliness to be a significant risk factor for human mortality from a wide variety of causes [8], an association observed across nations and populations at varying economic levels, from low to high income [9].

The documented toll on physical, as well as emotional, well-being is extensive. Evidence across 65 years of research consistently links social isolation and loneliness to worse cardiovascular and mental health outcomes [10], with health risks comparable in magnitude to hypertension, smoking cigarettes, and obesity [3,11]. Social isolation in childhood has been associated with poor cardiovascular health in adulthood, independent of other risk factors [12]. Lonely, isolated individuals suffer higher rates of cognitive decline and depression [8], and loneliness in older adults serves as a predictor of functional decline and death [13]. This physical toll may be mediated through its adverse impact on stress hormones, proinflammatory changes, and altered immunity [1].

## 3. Vulnerable Populations and the Impact of COVID-19

In 2020, the COVID-19 pandemic prompted an unprecedented exacerbation of social isolation, with global mandates for quarantines, social distancing, and the closure of schools, workplaces, houses of worship, and other institutions. Concerns arose immediately about the impact on the well-being of vulnerable populations such as children, adolescents, older adults, and those with preexisting mental health challenges. Researchers have already begun seeking to quantify and characterize the impact of these essential mitigation strategies on loneliness [14].

A growing body of research has already indicated high levels of distress and loneliness across various societies as life changed profoundly under pandemic restrictions, although accurate comparisons with pre-pandemic rates are more difficult to establish [15]. From January to September 2020, the numbers of people using an online screening tool offered by Mental Health America for anxiety and depression rose sharply, and more people reported frequent thoughts of suicide and self-harm than ever previously recorded in the Mental Health America program since its launch in 2014. Among people who screened with moderate–severe symptoms of anxiety or depression, 70% reported loneliness or isolation as one of the top contributors [16].

Confirmatory evidence for these intuitive findings is abundant. Early in the lockdown period, a large-scale assessment of US adults revealed “high loneliness” scores among 43% of respondents, correlating positively with higher rates of depression and suicidal ideation. That figure continued to rise to nearly 50% in subsequent months, despite partial reopenings—a paradox the investigators attributed to a “new normal” of profoundly altered social behaviors [17]. COVID-19 intensified concerns about isolation and loneliness among older adults, particularly those with medical or cognitive frailty [18], and among young people and those with mental health issues [15]. The pandemic also disrupted healthcare access, including mental health support delivered through schools [14]. Globally, there is evidence that caregivers and survivors of COVID-19 have also faced stigma in some settings, potentially prolonging and worsening social isolation and distress [19].

## 4. The Human–Animal Bond: Lifesaving Connection

Loneliness has been described as a biological signal to renew the connections needed to survive and prosper [1]. What do we understand about the ability of HAI to strengthen those connections, alleviate loneliness, and protect against its associated harms, both in “normal” times and unprecedented ones?

Pre-pandemic research on the potential health benefits of pets and companion animals (terms that will herein be used interchangeably) has focused on HAI as a source of social and emotional support, and as a catalyst for social interaction. People turn to their pets for “safe havens and secure bases” in the framework of attachment theory, which extends from parent–child interaction to other bonds of affection [20,21,22]. Undeniably, animals and humans may serve as attachment figures for one another [23], with attachments to pets demonstrated to rival or exceed those to people [21].

### 4.1. Evidence for Health Benefits of Companion Animals

Decades of research attest to the mutual bond between humans and companion animals as a source of comfort and reassurance [21], with benefits including better physical fitness, higher self-esteem, and social support comparable to that provided by parents or siblings [24]. Human–animal interaction has shown value, whether through pet ownership or animal-assisted therapy (AAT), in managing mental health issues, and offering affection, distraction, and an enhanced sense of control, security, and routine [25,26]. Importantly, many of these benefits are also shared by companion animals [21,23]. Proposed pathways for these effects may include neurochemical responses such as increases in oxytocin, dopamine, and endorphins, which have been observed in both humans and dogs when they interacted positively with one another [23].

Epidemiologic and longitudinal studies also show a positive association between pet ownership and physical health [23,27]. Individuals with companion animals have been shown to have fewer physician visits than those without pets [28]. In a seminal 1980 study, patients with acute coronary heart disease who owned a pet were significantly more likely to be alive a year after hospital discharge than non-pet owners, an effect confirmed in a larger sample of patients with cardiovascular disease in 2011 [29,30].

### 4.2. Animal Interaction and Social Capital

In the struggle against loneliness, at least during non-pandemic times, companion animals may also facilitate human interaction as social “icebreakers.” Being accompanied by a dog has been linked to more frequent social interactions, and increases the likelihood of being trusted and receiving help [23,31]. Service dogs substantially reduce the tendency of able-bodied people to ignore or avoid people with disabilities [32]. Such neighborhood interactions have even been proposed to have a ripple effect beyond pet owners, helping build a sense of community [33]. This HAI effect has been characterized as building social capital, broadly defined as the everyday connections that hold society together. In a study of three US cities and one Australian one, owning a pet was significantly associated with higher social capital, an association not confined to dog owners [34].

The evidence base for these positive effects of HAI on human well-being is not uniformly robust. Although HAI has been shown to correlate to better health in the majority of studies, some have yielded null results, and there is an overall need for larger, randomized controlled trials to document a causal relationship more firmly. Notably, intervention studies conducted with AAT offer stronger evidence for a positive impact of HAI on physical and emotional health [35].

## 5. Pets and the COVID-19 Pandemic

Media reports of surging pet ownership in developed nations, including fostering and adoption, during the COVID-19 pandemic appear to be supported by market research and other data [36]. In the United States, 67% of households—or almost 85 million households—own a pet, up from 56% in 1988, according to yearly data released by the American Pet Products Association, the leading pet products trade organization [37]. The year-over-year rate of growth accelerated in 2020 to 8% from about 3% over the past decade [38].

Whether newly acquired or not, companion animals in 2020 demonstrated the power of HAI to assuage loneliness at a historic moment of mass social isolation. In surveys in both the United States and the United Kingdom, pet owners reported that their animals, regardless of species, increased their well-being, and reduced stress and anxiety levels, and that the extra time with their pets strengthened the human–animal bond [39,40,41,42].

In “Pets in a Pandemic,” a survey of some 1000 pet owners by Mars Petcare—a leader in pet health products and research—86% of respondents cited companionship as a key benefit of animal companionship under COVID-19, 78% cited reduced stress or anxiety, and about three-quarters credited pets for reducing boredom and monotony, and conferring a sense of hope. One-third of pet owners surveyed had obtained a new pet in 2020, with more than half specifying “companionship” as their reason [43]. Regardless of the reasons for reported increases in animal companionship, as the pandemic changes to its next phase of altering lifestyles and routines, there are concerns that this may adversely impact companion animals, putting them at greater risk for stresses due to separation and potentially rehoming. Further research into the impact of these transitions on the bond will likely hold significant insights, which can be used to better support animal and human well-being.

## 6. Modeling Leadership by Building and Developing Consortiums: Implications on Human–Animal Interaction and Loneliness

The role of the human–animal bond in fostering healthy individuals and communities, particularly in a post-pandemic world, clearly merits further study and action, including policy change to support animal companionship among vulnerable populations. However, how can we bring this issue into focus and advance positive action? On a global scale, healthcare leaders across various disciplines have found high value in fostering change through initiatives such as health policy analysis institutes (“think tanks”) and other collaborative platforms, partnerships, and summit-type events. Key factors for success in influencing practice and policy have been identified, including: autonomy; allowing for critical and long-term perspectives; timely and relevant findings; the production of credible, trustworthy reports; personal connections among thought leaders and policy makers; and the presentation of actionable recommendations [44,45].

### 6.1. Establishing Mission and Partnership

Based on the success of these models, we sought to create an innovative and effective platform for leadership around HAI and the epidemic of social isolation and loneliness in the developed world. Human–animal interaction is an inherently interdisciplinary field, impacting widely varying dimensions of human health and well-being, making the consortium approach particularly well suited to facilitate positive change. In 2018, the Human Animal Bond Research Institute (HABRI)—a not-for-profit research and education organization that funds scientific research on the benefits of the human–animal bond—and Mars Petcare established the Consortium on Social Isolation and Companion Animals, a partnership to advance HAI in this context.

The partnership aimed not only to elevate awareness, research, and practice, but to also promote scientific rigor and ensure the welfare of the animals involved. People who experience and treat social isolation, and professionals who encounter its human toll in their work, were identified as key stakeholders. In May 2019, less than a year before the COVID-19 pandemic brought loneliness and the human–animal bond into sharp focus, HABRI and Mars Petcare cosponsored the first-ever Summit on Social Isolation and Companion Animals in Washington, DC. The summit engaged diverse experts and stakeholders to advance scientific research, share best practices, and overcome societal barriers to companion animals and AAT as assets in addressing loneliness. As a model for helping forge societal change around the human–animal bond through multidisciplinary leadership, this event merits a closer look at its creation, outcomes, and ongoing work [46].

### 6.2. Background

Leadership initiatives to promote HAI research and practice across the public and private sectors are not unprecedented. Beginning in 2008, a partnership between the Eunice Kennedy Shriver National Institute of Child Health and Human Development and the WALTHAM Petcare Science Institute laid some foundations for the current initiative. This groundbreaking project focused on the impact of HAI on child development and health, animal-assisted interventions (AAIs) for children and adults with disabilities, and the cost-effectiveness of HAI for public health. To date, this ongoing partnership has resulted in nearly 40 National Institutes of Health grants in HAI research, along with book and journal publications, workshops, and panels [46].

The support of the broader pet care community for HAI research through HABRI has also proven to be an effective model, with >40 pet care companies and organizations supporting HABRI’s research program. Since 2010, when HABRI was formed, this program has funded 35 research projects and a comprehensive online library of HAI research material housed at the Center for the Human–Animal Bond of Purdue University School of Veterinary Medicine.

### 6.3. Preparation: Steering Committee and Market Research

The first step in developing a summit event for the Consortium on Social Isolation and Companion Animals was the recruitment of an initial steering committee composed of multidisciplinary experts in various aspects of HAI. The committee summarized existing research with an eye to quality, and identified unmet needs and research questions, along with potential funding sources and opportunities for collaboration. Committee members prioritized focus areas for the summit agenda and identified key stakeholders to invite as summit event speakers and participants from fields including public health, research, psychology, gerontology, nursing, veterinary medicine, and AAT.

Before the summit in May 2019, the steering committee sought fresh information to help scope the issues and provide data-driven points to guide discussion on potential research topics and programmatic next steps. To address this need, HABRI and Mars Petcare commissioned a nationally representative market research project aimed at gaining a deeper understanding of the impact of companion animals on social isolation and loneliness. The market research was a 30-minute online questionnaire conducted in the United States among 2036 respondents, including 1469 pet owners [47]. Findings from the survey are presented in Table 1. These results validated the steering committee’s premise that the human–animal bond can play a role in alleviating social isolation and loneliness. The following year, their efforts bore fruit in the Summit on Social Isolation and Companion Animals.

### 6.4. Summit Goals and Programming

The Consortium on Social Isolation and Companion Animals’ steering committee laid out the following goals for the Summit on Social Isolation and Companion Animals:

- Engage experts and stakeholders in establishing the role of companion animals in helping to prevent, mitigate, and/or alleviate social isolation across the age spectrum;
- Articulate key questions for further study to validate the benefits of HAI in this role;
- Identify barriers to realizing the benefits of HAI, particularly for people vulnerable to social isolation; and
- Propose best practices and practical solutions to address these barriers.

The summit succeeded as a mission-building conversation across disciplines and the public/private spheres. Vivek H. Murthy, MD, 19th Surgeon General of the United States, highlighted the foundational need for personal connection in a healthy society, with animal interaction in a range of settings as a potential way to spark such connection. Presenters and panelists, representing a remarkable breadth of disciplines in human and animal health and welfare, focused on two vulnerable populations: older adults and people with mental health challenges. A full program of topics and speakers, along with highlights of the summit outputs, are available at: https://habri.org/assets/uploads/Addressing-the-Social-Isolation-and-Loneliness-Epidemic-with-the-Power-of-Companion-Animals-Report.pdf (accessed on 11 June 2021).

## 7. Future Vision from the Consortium: Less Isolation, More Animal Companionship

The Consortium on Social Isolation and Companion Animals’ initial summit solidified a commitment by HABRI and Mars Petcare, which was influenced by the proceedings and the interchange of ideas towards implementing three action-oriented goals:

*Advance high-quality HAI research.* Priorities include going beyond the pet-owner/nonowner binary to include qualitative data about the human–animal bond, and improving methodology to include standardized measures, randomization, large and diverse samples, and, whenever possible, longitudinal designs to illuminate the long-term effects of animal interaction (Table 2 presents a synopsis of recommendations). Research and practice around AAI should aim for evidence-based protocols that may be shared and replicated, with diverse participants.

*Share and support best practices*. These practices promote the safety and success of AAI in hospitals, nursing homes, and other settings. Pillars of this goal include high standards for animal welfare safeguarded by the involvement of veterinarians and animal behaviorists, scaling up and sharing learnings from effective programs, and ongoing monitoring.

*Address barriers and provide solutions.* Action items include research funding and direction; support for potential pet adopters to responsibly select and care for animal companions; policy development for pet and AAI access in housing, public spaces, and healthcare settings; education of clinicians and care teams; and elevating the role of companion animals and AAI through outreach and communication.

### 7.1. Concrete Outputs of the Consortium and Summit

The Summit on Social Isolation and Companion Animals was a meeting of minds and a call to action, but its most valuable outcomes were the wealth of activities that followed. After the event, the steering committee and several summit participants formed ongoing working groups to advance the Consortium on Social Isolation and Companion Animals’ identified goals. Members of the working groups include the original steering committee participants, summit attendees, and representatives from HABRI and Mars Petcare. The two working groups each focused on a major consortium area of concern, i.e., older adults and people with mental health challenges. Convening virtually, the groups have been tasked with advancing best practices in HAI and pet ownership, as well as formulating ideas for future research and funding to advance HAI scientific study. In the short and turbulent interval since the 2019 summit, their efforts have already paid off in several initiatives:

- A report summarizing the goals and presentations of the May 2019 summit was created and shared with various stakeholders and the general public. Working group members reviewed and contributed to this report and assisted in sharing it widely with their respective organizations and networks.
- A research survey project has been undertaken with the Humane Rescue Alliance in Washington, DC, on the experiences and attitudes of people who adopted or fostered a new pet between March and November 2020. Preliminary findings indicate that companion animals had a profound impact on humans during this historically isolating period, and that resources for animal care and behavior management (i.e., training) should be made available to keep animal companions in the home and preserve this valuable bond.
- The working groups developed a study protocol for a novel randomized, controlled pilot study of AAI in older adults undergoing inpatient rehabilitation. The study, funded by Mars Petcare at Virginia Commonwealth University School of Medicine’s Center for Human–Animal Interaction, will compare animal intervention with a conversational control (a handler without a dog) and treatment as usual. This longitudinal study aims to assess the effectiveness of therapy-dog interventions in improving loneliness and health-related outcomes for patients, as well as exploring the well-being of the dogs involved, with completion expected by 2023. The results will help inform the implementation and feasibility of future large-scale, multicenter clinical trials.
- Working group members have shared findings and insights through presentations to conferences in the veterinary medicine and anthrozoology spaces [48,49], and through journal publications [50,51].

### 7.2. Synergy as a Driver of New Initiatives

Continued engagement by the Consortium on Social Isolation and Companion Animals’ working groups in coming years will be aimed at developing further events, publications, congress presentations, and digital resources to make HAI more accessible and effective for people suffering from social isolation and loneliness, while advancing the welfare of companion animals. The group’s ambitious vision is to usher in a wave of new opportunities to facilitate HAI for people who may benefit most.

**Figure 1:**
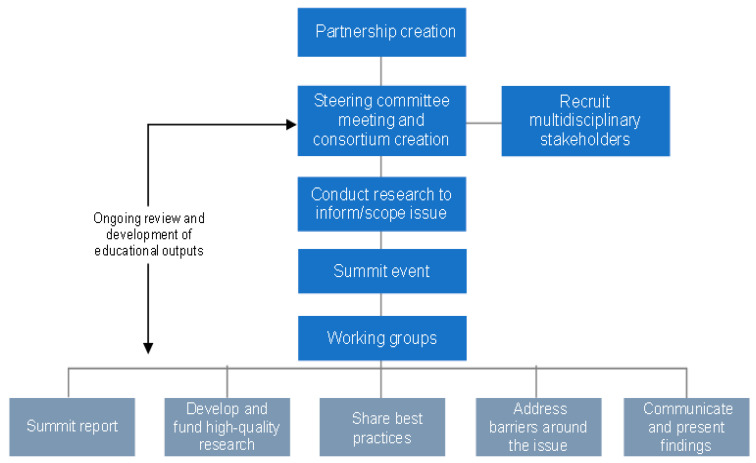
Key steps in development and implementation of a leadership consortium.

Importantly, those involved in this partnership recognize the potential of the consortium model to create cross-disciplinary synergy that may branch off into other areas of research and endeavor around the human–animal bond. It is hoped that the consortium and summit experiences continue to help inspire and connect others to move forward together within or outside the structures that have been created and nurtured. An overview of key development steps and potential outcomes of a leadership consortium modelled on the HABRI/Mars Petcare collaboration is presented in Figure 1.

## 8. Conclusions: Building New Vehicles for Change

The Consortium on Social Isolation and Companion Animals may serve as a model for other innovative partnerships in the field of HAI seeking to elevate the joint welfare of animals and humans. Critical factors for the success and effectiveness of such initiatives are summarized in Table 3. One key from the outset was to enable conversations across many sectors, including science, academia, medicine, tech, nonprofit, and industry. Another key was to focus on a specific area of unmet need—in this case, loneliness—through multiple lenses, and to include diverse stakeholders at the table and amplify their voices.

The timeliness of this consortium’s commitment to advancing HAI for loneliness is striking. At the consortium’s 2019 kickoff event, no one could have foreseen the societal transformations just ahead in the COVID-19 pandemic. Integrating animal companionship into the uncertainties of a post-pandemic world raises challenges and possibilities, including broad social shifts in expectations of where companion animals are welcome in our communities and around issues such as work/life balance and pet-friendly workplaces. In a recent survey by Banfield Pet Hospital, 57% of respondents said they would be happiest returning to work if they could bring their pets with them and employers are increasingly open to new pet-friendly policies [52].

This inflection point in appreciation for HAI comes on the heels of rapid expansion in the field at large. Over the past decade, at least seven new centers of excellence have been established at academic institutions, including the Tufts Institute for Human–Animal Interaction, the Human–Animal Interaction Research Initiative at the University of Arizona, and the Center for Animal Human Relationships at Virginia Polytechnic Institute and State University. Research funding has also risen, with the establishment of HABRI and additional streams of funding including from the National Institutes of Health, Mars Petcare/WALTHAM Petcare Science Institute, and Nestlé Purina [53]. Human–animal interaction is increasingly recognized at scientific conferences and the bar for research has already been raised, including more attention to the effects of HAI on animal participants.

The COVID-19 pandemic has laid bare not only the toll of loneliness, but many underlying inequities in financial security, access to health care, and social capital. Lockdowns and social distancing have also prompted a new appreciation of the human–animal bond, even as many people most vulnerable to social isolation, before and throughout the pandemic, face barriers to its consolation and connectedness. To meet these challenges, we will continue to nurture innovative partnerships, using the most successful strategies of platforms such as “think tanks” and global leadership summits to advance the joint welfare of humans and animals.

## Figures and Tables

**Table 1:** Findings of a market research study on the impact of companion animals on social isolation and loneliness among 2036 US respondents, including 1469 pet owners (72%) [47] *.

80% of pet owners reported that their pet makes them feel less lonely.
51% of pet owners said their pet helps them feel less shy.
Among both pet owners and non-pet owners, 85% of respondents believed interaction with a companion animal reduces loneliness and 76% agreed that human–animal interactions can help address social isolation; among pet owners, those with the closest bond to their pet saw the highest positive impact on feelings of loneliness and social isolation.
54% of respondents said their pet helped them connect with other people.
89% of pet owners who adopted a pet due to loneliness believed their pet helped them feel less lonely.
26% of pet owners reported having adopted a pet because they believed it was good for mental health; respondents aged > 55 y reported doing so more frequently.

* UCLA Loneliness Scale and Monash Dog-Owner Relationship Scale were used in analysis to explore key themes, with data tested at 95% confidence interval.

**Table 2:** Recommendations for HAI research advancement generated by participants at the Summit on Social Isolation and Companion Animals held in Washington, DC, in May 2019.

Research Quality
Establish standardized and well-validated outcome measures
Design studies with rigorous methodology (comparison groups, sample sizes)
Describe methodology adequately to permit replication
Elevate diversity in research populations and access to programming
Research questions:
Include qualitative as well as quantitative measures of HAI (beyond “pet/no pet” binary to include relationships and interactions)
Consider unmet needs of underserved communities and vulnerable populations
Explore underlying mechanisms for documented benefits
Study the impact of HAI on the animals and their well-being
Follow-up activities:
Share data from smaller trials to enrich resources for the research community
Move research outcomes into practice, via specific, evidence-based guidance for specific populations
Develop clear, informative messaging for stakeholders to leverage research findings for maximum benefit
In older adults:
Examine the role of animal companionship in navigating life transitions such as retirement or loss of a spouse
Plan longitudinal studies to explore the value of pets to healthy aging
In people with mental health challenges:
Elucidate optimal circumstances for HAI, including animal-assisted therapy
Evaluate the efficacy of interventions, including patient satisfaction
Identify differences in HAI for this population and their implications in practice
HAI, human–animal interaction.

**Table 3:** Critical factors for the planning and execution of effective leadership initiatives based on the success of the Summit on Social Isolation and Companion Animals.

Recruit a diverse, multidisciplinary group of experts to form an advisory or steering committee. Their task is to prioritize focus areas for a larger consortium effort and identify key stakeholders to invite as participants. This committee can help develop the agenda and suggest speakers for a summit event and participate in the creation of outputs such as a summary report.
Develop a communication plan to promote the event to the public, possibly with the professional help of a public relations team. Consider using interview content from event participants for earned or social media exposure. Even post-event promotion helps raise awareness and heightens interest in its goals and outcomes.
In choosing a date for an event, keep in mind months of recognition or special awareness holidays, e.g., HABRI/Mars Petcare held the 2019 summit in May, during Mental Health Month.
Consider offering sponsorship opportunities for certain stakeholders to offset costs while also raising the profile of the event.
With the steering committee/advisory group recommendations in mind, invite a broad group of stakeholders to attend the summit event. Plan to include speaker Q&As, discussion groups, or other means of connecting and engaging event participants with one another.
Ask the steering committee to brainstorm outcomes and key takeaways from the event, and request that they come prepared with next steps or direct “asks” for the audience to consider.
After the event, create an action plan that involves participants, the steering committee, and others. Before the HABRI/Mars Petcare summit, plans were laid out to create and disseminate a report summarizing the event and the consortium’s overall effort, including a detailed account of the recommendations borne out of the discussions.
Offer summit event participants the opportunity to join working groups and encourage ongoing service by steering committee members to ensure continuity. Our working groups were tasked with promoting the consortium’s goals of advancing research, sharing best practices, and addressing barriers. HABRI and Mars Petcare have continued to engage and support working-group members with invitations to present on the consortium’s efforts and participate in other relevant conferences.
Consider the areas of expertise of steering committee members and participants: Are they researchers, policy makers, or service providers? HABRI and Mars Petcare balanced each working group to include researchers and practitioners from varied backgrounds so that each individual could bring something unique to the table.
HABRI, Human Animal Bond Research Institute.
